# Cone-Beam Computed Tomography (CBCT)-Guided Adaptive Boost Radiotherapy for a Patient With Locally Advanced Cervical Cancer Ineligible for Brachytherapy

**DOI:** 10.7759/cureus.66218

**Published:** 2024-08-05

**Authors:** Alice E Silberstein, Joshua P Schiff, Robbie Beckert, Xiaodong Zhao, Eric Laugeman, Stephanie Markovina, Jessika A Contreras

**Affiliations:** 1 Department of Radiation Oncology, Washington University School of Medicine, St. Louis, USA

**Keywords:** online adaptive radiation therapy, locally advanced cervical cancer, brachytherapy ineligible, cbct-guided adaptation, cone-beam computed tomography (cbct)

## Abstract

Brachytherapy is a critical component of locally advanced cervical cancer treatment, and patients ineligible for brachytherapy historically have poor outcomes. Delivery of boost with stereotactic body radiation therapy (SBRT) has been studied, though toxicity is a concern. Recent case reports have explored adaptive radiation boost, which can adjust plans for inter-fraction motion using magnetic resonance guidance. Herein, we report the first patient with locally advanced cervical cancer ineligible for brachytherapy who was treated with a cone-beam computed tomography (CBCT)-guided adaptive boost following completion of chemoradiation. A 71-year-old female with locally advanced cervical cancer was treated with chemoradiation and was deemed ineligible for a brachytherapy boost due to tumor size, geometry, and a fistula with a tumor in the bladder. She was prescribed a boost to the primary tumor of 25 Gy in five fractions using CBCT-guided adaptive radiation following the completion of chemoradiation. A simulation was performed using a non-contrast CT fused with a mid-chemoradiation magnetic resonance imaging (MRI) scan to create an initial plan. For each treatment fraction, kilovoltage CBCTs were acquired, contours of organs at risk (OARs) were adjusted to reflect anatomy-of-the-day, and an adapted plan was generated. The initial and adapted plans were compared using dose-volume histogram objectives, and the adapted plan was used if it resolved OAR constraint violations or improved target coverage. The use of the initial treatment plan would have resulted in constraint violations for the rectum, sigmoid, and bladder in all fractions. The adapted plans achieved hard constraints in all fractions for all four critical OARs. The mean total treatment time across all five fractions was 58 minutes. This case demonstrates the feasibility of a CBCT-guided adaptive boost approach and the dosimetric benefits of plan adaptation in this setting. Though larger-scale and longer-term data are needed, CBCT-guided adaptive radiation may present a feasible alternative modality to deliver boost doses for brachytherapy-ineligible patients.

## Introduction

Radiation therapy with concurrent chemotherapy is the standard treatment for locally advanced cervical cancer [1,2]. Brachytherapy, used to deliver a boost to the primary tumor, is a critical component of definitive radiation in this setting. However, patients may be ineligible for brachytherapy for various reasons, including anatomic limitations, medical comorbidities, or refusal to undergo brachytherapy [3-5], and such patients with contraindications to brachytherapy or severe medical conditions have been excluded from the landmark EMBRACE I/II trials [6,7]. Treatment of these patients is challenging: population-based studies repeatedly demonstrate inferior survival among those treated without brachytherapy [8-10], and studies delivering boost doses using external beam radiation therapy (EBRT) alone in this population historically have resulted in poor local control and survival outcomes [3,11-13].

More recently, alternative approaches to boost delivery for brachytherapy-ineligible patients have been explored. In particular, there has been interest in the use of stereotactic body radiation therapy (SBRT), and several studies have reported improved local control compared to historic EBRT boost studies [4,5,14-16]. However, toxicity is a concern, given the potential for the movement of cervical tumors and organs at risk (OARs), which may not be well visualized on standard cone-beam computed tomography (CBCT) scans [4,17]. Adaptive radiation is well suited to address the challenges of inter-fraction motion [18], and select case reports have utilized magnetic resonance-guided adaptive radiation for brachytherapy-ineligible patients [19-21]. Herein, we report the first patient with locally advanced cervical cancer ineligible for brachytherapy who was treated with a CBCT-guided adaptive boost following the completion of chemoradiation.

## Case presentation

Patient presentation

**Figure 1:**
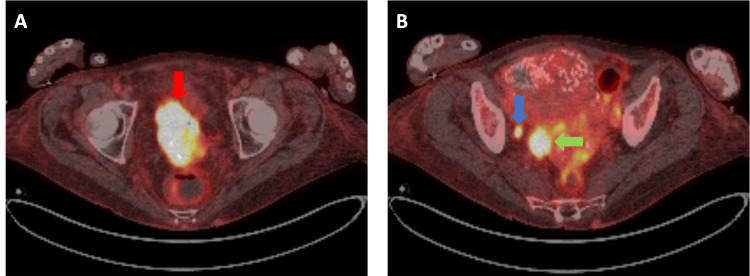
Pre-treatment PET-CT This patient had IVA disease, including a bulky primary tumor invading the bladder (red arrow, panel A) as well as pelvic node (blue arrow, panel B) and right ovarian (green arrow, panel B) metastases, amongst other clinical findings. PET-CT: Positron emission tomography-computed tomography

**Figure 2:**
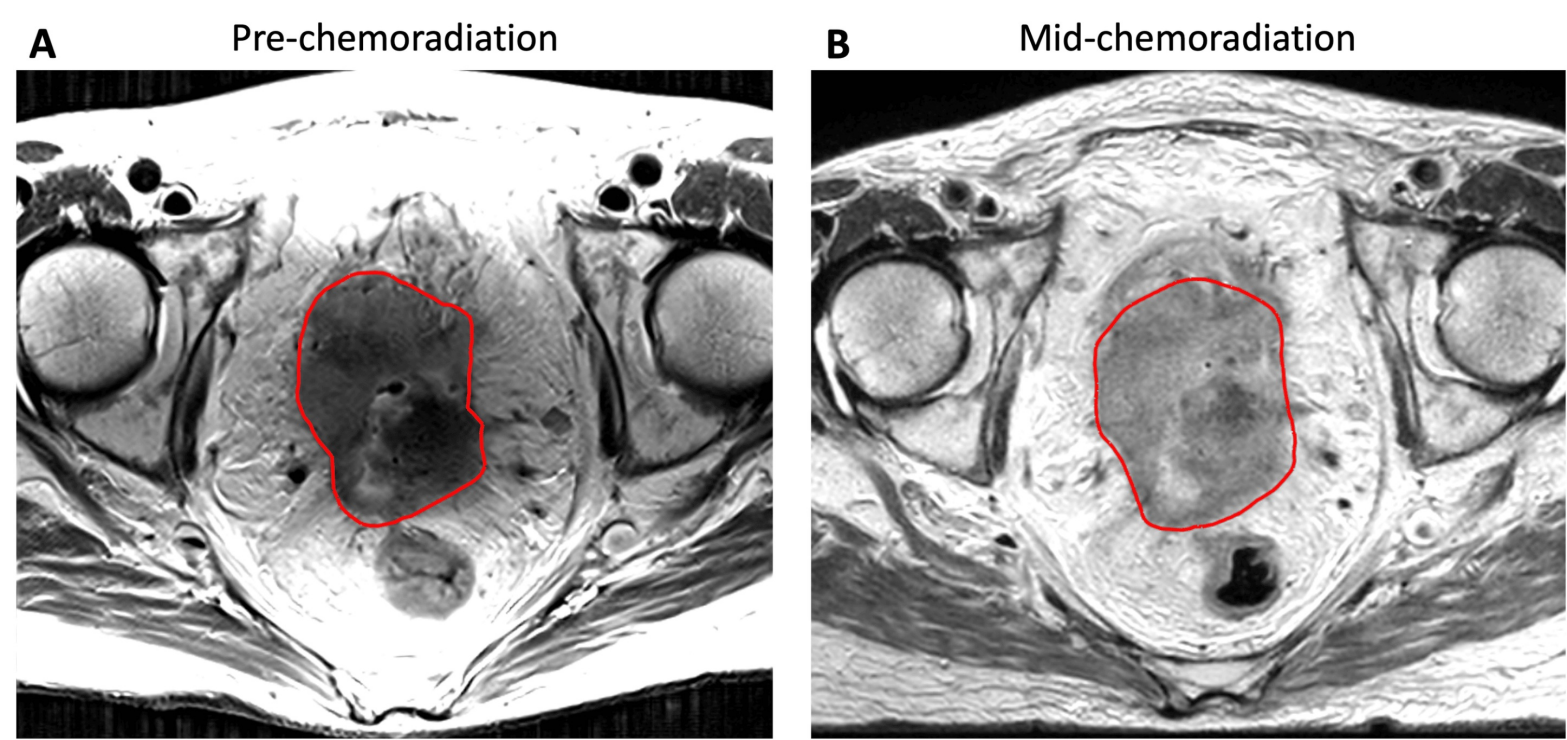
Pre- and mid-treatment T2-weighted MRIs Diagnostic MRI of the tumor pre-treatment (panel A) and MRI of the tumor midway through chemoradiation, prior to boost delivery (panel B), with a red outline indicating the contours used to delineate tumor extent in planning initial external beam (panel A) and boost (panel B) treatments. This patient had minimal response to chemotherapy and external beam radiation. Note the bulky, largely anterior disease, which made this patient ineligible for brachytherapy per the treating gynecologic radiation oncologist. MRI: Magnetic resonance imaging

A 71-year-old woman presented to the hospital with vaginal bleeding and renal failure. During her work-up, imaging demonstrated a 6.8 cm infiltrative cervical mass invading the urinary bladder, upper vagina, and bilateral ureters, as well as metastases to pelvic nodes, para-aortic nodes, and the right ovary (Figure 1). A vesicovaginal fistula was also present. A biopsy of the cervical mass confirmed squamous cell carcinoma. Due to ongoing vaginal bleeding requiring repeated transfusions, the patient first received a palliative course of cervical ring brachytherapy consisting of 10 Gy in two fractions prescribed to the surface of the cervix; this is our institutional standard for emergent vaginal bleeding due to cervical tumors and has been previously published [22]. Per departmental workflow, we do not include this dose in our overall EBRT/boost dose calculations. For definitive treatment, she was recommended EBRT with concurrent weekly carboplatin, followed by a boost to her primary tumor. EBRT consisted of 50.4 Gy in 28 fractions to her primary mass, pelvic nodes, and para-aortic nodes, with simultaneous integrated boosts to the right ovary (55 Gy) and positron emission tomography (PET)-positive para-aortic and common iliac nodes (60 Gy). Midway through the patient’s course of EBRT, pelvic exam and magnetic resonance imaging (MRI) were repeated and demonstrated persistently bulky and largely anterior disease (Figure 2). Given the tumor size, geometry, and the fistula with a tumor in the bladder that was not accessible with an applicator or interstitial needles, she was deemed ineligible for brachytherapy. Instead, she was recommended for a boost treatment using CBCT-guided adaptive radiation, consisting of 25 Gy in five fractions.

Boost treatment planning and delivery

An additional simulation was performed after the completion of chemoradiation to redefine tumor anatomy and to create an initial plan (P_i_) for the boost treatment. The patient was simulated using a non-contrast CT. She was positioned supine, with her arms on her chest and without immobilization. Of note, the patient had bilateral percutaneous nephrostomy tubes in place and, therefore, had a consistently empty bladder. Fiducials were placed prior to any treatment to delineate tumor extent, and the MRI obtained midway through the patient’s EBRT course was fused to the simulation images to assist in target delineation.

**Figure 3:**
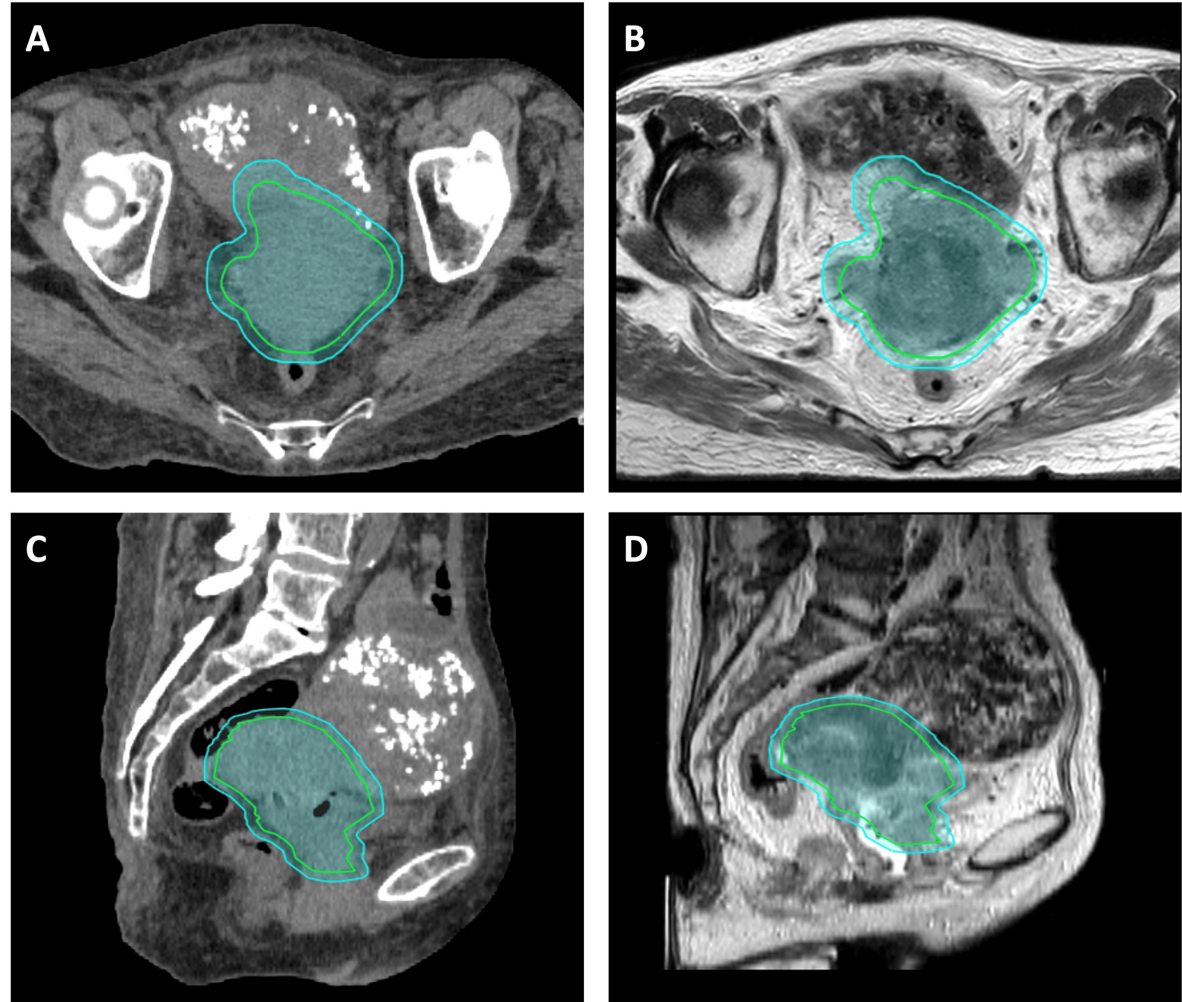
Boost plan target contours CTV (green) and PTV (cyan) are depicted on both CT (left, panels A and C) and MRI (right, panels B and D) simulation on axial (top, panels A and B) and sagittal (bottom, panels C and D) slices. Note that the patient has a fibroid uterus, which was not included in the CTV per our institutional standard. CTV: Clinical target volume; PTV: Planning target volume; CT: Computed tomography; MRI: Magnetic resonance imaging

All planning was performed in the ETHOS (v.1.1) treatment planning system (TPS). The gross tumor volume (GTV) comprised the gross tumor on simulation CT and T2-weighted MRI. The clinical target volume (CTV) was defined as the GTV plus the entire cervix, any areas of presumed extra-cervical tumor spread, and T2 MRI gray zones, according to high-risk CTV volume delineation, as described in the EMBRACE II protocol (Figure 3) [7]. Of note, our high-risk CTV volumes do not include the entire uterus [23]. A 0.7 cm uniform volumetric expansion was applied to the CTV to form a planning target volume (PTV).

OARs, namely the bladder, rectum, sigmoid, and bowel, were contoured on axial slices from 3 cm below to 3 cm above the PTV. Dose constraints are defined in Table 1. To establish these constraints, the maximum dose to OARs was calculated from the EBRT plan and converted to the biologically equivalent dose in 2 Gy per fraction (EQD2_3_) using an alpha/beta ratio of 3 for normal tissue. Per-fraction constraints were then calculated by subtracting the EQD2_3_ dose delivered with EBRT from standard widely accepted cervical cancer constraints [2,7]. Of note, we did not use the EMBRACE II D2 cc metrics for the boost constraints, as this was an SBRT-like plan, and we were primarily concerned with limiting maximum doses to minimize toxicity. Therefore, we converted the constraint to volumetric 0.03 cc constraints. In keeping with institutional adaptive radiation practices [24-26], maximum volumetric 0.03 cc constraints were prioritized over target coverage. One exception to this approach was in the area of gross tumor invasion into the bladder with a pre-existing fistula, where target coverage was prioritized. To limit dose in other areas of direct overlap between the PTV and OARs, a PTV optimization structure was created by taking the PTV and subtracting the OARs with the addition of a margin (5 mm for sigmoid and rectum, 3 mm for bladder). This structure was then used to drive prescription doses during treatment planning.

**Table 1:** OAR dose constraints The boost D2 cc dose constraints for the critical OARs were derived using EQD2_3_ calculations based on maximum OAR tolerances as dictated by the EMBRACE II protocol. Once the max EQD2_3_ EBRT contribution was calculated per OAR, the remaining dose was converted into a volumetric constraint for the five-fraction boost plan. OAR: Organ at risk; EBRT: External beam radiation therapy; EQD2_3_: Biologically equivalent dose in 2 Gy per fraction, using an alpha/beta ratio of 3

OAR	EQD2_3_ D2cc constraint	EBRT Dmax	EBRT EQD2_3 _Dmax	Boost tolerance dose per fraction (for a five-fraction plan)	Boost volumetric constraint (V 0.03 cc ≤)
Bladder	90 Gy	54 Gy	53.2 Gy	5.0 Gy	25 Gy
Rectum	75 Gy	54 Gy	53.2 Gy	3.4 Gy	17 Gy
Sigmoid	75 Gy	54 Gy	53.2 Gy	3.4 Gy	17 Gy
Bowel	75 Gy	60 Gy	61.7 Gy	2.4 Gy	12 Gy

An adapted plan was created for each treatment fraction based on the patient’s anatomy-of-the-day using a daily kilovolt (kV) CBCT. The TPS then auto-contoured the bladder and rectum OARs using an artificial intelligence algorithm. Sigmoid and bowel contours were deformably registered and then manually edited by the treatment team. The simulation GTV and CTV were rigidly copied over to the anatomy-of-the-day and edited at the discretion of the covering radiation oncologist. Subsequently, expansions to generate the PTV were reapplied. The P_i_ was projected onto this anatomy-of-the-day, and simultaneously, a re-optimized adapted plan (P_a_) was generated. The plans were compared using dose-volume histogram (DVH) objectives, and the P_a_ was used if it resolved an OAR hard constraint or improved target coverage by 5% or greater when compared to the P_i_.

If the P_a_ was selected for treatment, a subsequent secondary dose calculation quality assurance software was run, and treatment was delivered. If the P_i_ was selected, no additional quality assurance was performed. A post-adaptive contouring and planning CBCT, as well as a mid-treatment CBCT, were acquired to confirm target positioning, and the treatments were delivered with intra-fraction free breathing motion monitoring using surface guidance.

Dosimetric and treatment data

**Figure 4:**
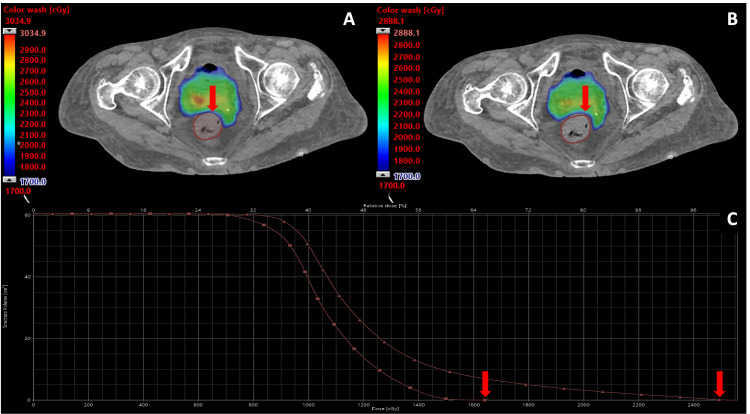
Comparison of initial and adaptive plans Initial (panel A) and adaptive (panel B) plans for a single fraction of radiation. In the initial plan, the high-dose color wash (>17 Gy) is in the rectum (red outline), while in the adapted plan, the high-dose color wash does not enter the rectum. The dose volume histogram (panel C) demonstrates the dose delivered to the small bowel in the initial (triangle) and adaptive (square) plans, with arrows labeling the maximum rectum dose for each plan.

PTV volume per fraction, in order of fractions, was 407.4 cc, 420.9 cc, 412.7 cc, 423.8 cc, and 420.1 cc, with a mean PTV volume of 417.0 cc across fractions. Constraint and coverage metrics for the P_i_ and P_a_ overlaid on anatomy-of-the-day are demonstrated in Table 2. In all five fractions, the use of the P_i_ on the patient’s anatomy-of-the-day would have resulted in bladder, rectum, and sigmoid constraint violations. In contrast, the P_a_ achieved constraints in all five fractions and was used for treatment every day. Figure 4 compares the P_i_ and P_a_ in a single fraction and illustrates how daily adaptation allowed this fraction to achieve the rectum hard constraint, which would have been violated if the P_i_ had been used.

**Table 2:** Target metrics and OAR constraints Target metrics and OAR constraints for each of the five fractions when the initial (P_i_) and adapted (P_a_) plans were overlaid on the anatomy-of-the-day. There were bladder, rectum, and sigmoid constraint violations when using the P_i_ in each fraction, resulting in a total of 15 dose constraint violations (bolded in the table) and the use of P_a_ for treatment every day. Note that the V100% is not always increased in the P_a_ compared to the P_i_, as in certain fractions target coverage may need to be sacrificed to meet an OAR constraint. * Represents dose to bladder excluding the bladder volume with gross tumor invasion and pre-existing fistula. PTV: Planning target volume; CTV: Clinical target volume; GTV: Gross tumor volume; OAR: Organ at risk

Parameter	Fraction 1		Fraction 2		Fraction 3		Fraction 4		Fraction 5	
	P_i_	P_a_	P_i_	P_a_	P_i_	P_a_	P_i_	P_a_	P_i_	P_a_
PTV V100% (%)	74.2%	30.2%	75.7%	67.7%	75.3%	62.1%	73.0%	60.7%	61.1%	64.4%
CTV V100% (%)	86.8%	45.8%	87.5%	82.1%	86.6%	78.1%	84.8%	77.6%	78.1%	79.3%
GTV V100% (%)	95.3%	82.3%	95.0%	94.5%	94.5%	94.4%	89.2%	89.8%	94.3%	95.5%
Bladder V25 Gy ≤ 0.03 cc (cc)*	0.67 cc	0.00 cc	1.18 cc	0.00 cc	0.47 cc	0.00 cc	0.09 cc	0.00 cc	0.16 cc	0.00 cc
Rectum V17 Gy ≤ 0.03 cc (cc)	6.02 cc	0.03 cc	7.00 cc	0.02 cc	11.70 cc	0.03 cc	9.18 cc	0.03 cc	7.80 cc	0.02 cc
Sigmoid V17 Gy ≤ 0.03 cc (cc)	4.56 cc	0.00 cc	9.85 cc	0.02 cc	1.10 cc	0.00 cc	2.52 cc	0.03 cc	2.06 cc	0.02 cc
Bowel V12 Gy ≤ 0.03 cc (cc)	0.00 cc	0.00 cc	0.00 cc	0.00 cc	0.00 cc	0.00 cc	0.00 cc	0.00 cc	0.00 cc	0.00 cc

Treatment component times were recorded and are demonstrated in Table 3. The mean total treatment time was 58 minutes. Over the course of chemoradiation and adaptive boost, the patient experienced grade 1 fatigue, grade 1 nausea, and grade 2 diarrhea, all of which resolved at six weeks post-treatment. The patient completed all treatments and is currently alive, without evidence of high-grade toxicity or local recurrence, at two months post-treatment.

**Table 3:** Treatment component times Treatment component times are presented for each fraction in minutes. Plan adaptation is measured as time from initial CBCT to pre-treatment CBCT, including time required for contouring, plan re-optimization, plan review, and quality assurance. Timing measurements do not include patient setup time. CBCT: Cone beam computed tomography

Treatment fraction	Plan adaptation time (minutes)	Beam delivery time (minutes)	Total treatment time (minutes)
Fraction 1	32	24	56
Fraction 2	33	13	46
Fraction 3	35	33	68
Fraction 4	42	22	64
Fraction 5	36	20	56
Mean	36	22	58
Standard deviation	3.9	7.2	8.5

## Discussion

In this case study, we describe the first reported patient with locally advanced cervical cancer ineligible for brachytherapy who was treated with a CBCT-guided adaptive boost following completion of chemoradiation. Our results illustrate that delivery of P_i_ would have resulted in constraint violations for the bladder, rectum, and sigmoid in each fraction, while P_a_ met all OAR constraints and simultaneously achieved excellent GTV coverage for each fraction. With this approach, the patient received 80.8 Gy EQD2_10_, using an alpha/beta ratio of 10 for the tumor, in total across chemoradiation and boost treatments without violating constraints for critical pelvic organs. Of note, we delivered a lower dose relative to the 85 Gy target in EMBRACE II [7] to prioritize the sparing of OARs, given that a prior study delivering SBRT in a similar setting resulted in grade 5 toxicities [4].

Brachytherapy is a vital component of cervical cancer treatment [1,2,27]. It offers key advantages over EBRT, which collectively facilitate the sparing of normal tissues: radiation sources with rapid dose fall-off are placed in close proximity to the tumor, and the securing of brachytherapy devices reduces uncertainty in the positioning of the tumor and OARs [28]. As a result, the brachytherapy boost allows for the delivery of high total doses of over 85 Gy (EQD2_10_) to tumors with low rates of toxicity [6]. Nevertheless, many factors may preclude patients from brachytherapy, and the delivery of sufficient doses is challenging in this population because the dose delivered via external radiation is limited by the tolerance of surrounding organs [11,12]. In prior studies delivering EBRT boost using 3D conformal radiation, lower total doses of 54-72 Gy were prescribed [3,11-13]. These studies consistently demonstrated poor outcomes, including high rates of incomplete response and local failure, as well as poor overall survival (Table 4). SBRT has since become an area of interest in this setting due to its ability to deliver highly conformal doses [28]. However, the inability to control motion, including the movement of critical normal structures and tumor regression during treatment [29], presents a key limitation for SBRT in this setting [17,18]. While a number of small studies have used SBRT to deliver higher total doses to tumors, with median EQD2_10_ ranging from 74 to 90 [4,5,14-16], toxicity is a concern: one key study closed prematurely due to GI toxicity, including two toxicities resulting in death [4]. As a result, the safe delivery of adequate boost doses remains a challenge in the brachytherapy-ineligible population.

**Table 4:** Summary of studies exploring boost alternatives among cervical cancer patients unsuitable for brachytherapy * Represent outcomes as of the patient’s last follow-up unless otherwise specified; ** 71% received 60-65 Gy; *** Represents LC among patients who achieved CR EQD2_10_: Biologically equivalent dose in 2 Gy per fraction, using an alpha/beta ratio of 10; CR: Complete response; LC: Local control; LRC: Locoregional control; PFS: Progression-free survival; OS: Overall survival; GI: Gastrointestinal; GU: Genitourinary; NR: Not reported; EBRT: External beam radiation therapy; SBRT: Stereotactic body radiation therapy; MR: Magnetic resonance; ART: Adaptive radiation therapy

Boost approach	Study	N	Median total dose Gy, EQD2_10_ (range)	Median follow-up (months)	Oncologic outcomes*				GI/GU toxicity (≥ grade 3)
					CR	LC/LRC	PFS	OS	
EBRT boost	Barraclough et al. (2008) [11]	44	NR** (54-70)	28	NR	64% LC	52%	49% at 5 years	2% acute, 2% late
	Kadkhodayan et al. (2013) [12]	30	70	26	63%	NR	33%	39% at 3 years	13% acute, 17% late
	Karlsson et al. (2017) [3]	86	68 (64-72)	42 (mean)	73%	83% LC***	25%	25% at 5 years	0% acute, 8.1% late
	Ito et al. (2019) [13]	37	60 (56-70)	17	50%	68% LC	29% at 2 years	43% at 2 years	14% acute, 5% late
SBRT Boost	Haas et al. (2012) [14]	6	84 (76-84)	14	100%	100% LC	100%	100%	0% total
	Hsieh et al. (2013) [15]	9	79 (65-83)	36	78%	78% LRC	26%	47% at 3 years	11% acute, 11% late
	Marnitz et al. (2013) [16]	11	90	6	100%	100% LC	100%	100%	0% total
	Albuquerque et al. (2020) [4]	15	84	19	NR	70% LC at 2 years	47% at 2 years	53% at 2 years	27% total
	Ito et al. (2024) [5]	21	74 (74-77)	40	95%	84% LRC at 2 years	67% at 2 years	81% at 2 years	5% acute, 5% late
MR-guided ART boost	Ugurluer et al. (2024) [21]	4	86 (82-90)	11	67%	NR	NR	75% at 1 year	0% total

Adaptive radiation therapy (ART) with MR or CBCT guidance may present a suitable solution for this patient population due to the ability to adjust plans to account for inter-fraction motion [18]. Adaptive planning is well-established in cervical cancer: image-guided adaptation is standard practice for cervical brachytherapy [30,31], and studies have demonstrated that adaptation in this setting improves dosimetry [32,33] and achieves excellent clinical outcomes [6,34]. To date, four cases have been reported of MR-guided ART boost following definitive chemoradiation for cervical cancer, and a median total dose of 86 Gy (range 82-90, EQD2_10_) was prescribed [19,21]. There are three other published cases where MR-guided ART was used to boost the primary tumor in gynecologic cancer: one for a cervical cancer recurrence, another for endometrial recurrence with bladder involvement [21], and another in which the patient received EBRT, two fractions of brachytherapy, and then MR-guided ART [20]. All aforementioned cases were delivered without violating critical OAR constraints, and no grade 3 or higher gastrointestinal or genitourinary toxicities have been reported. Though the number of cases is limited and long-term follow-up data are required, these data provide preliminary support that ART may be a feasible option to provide a boost dose when patients are ineligible for brachytherapy.

Our case report similarly describes the delivery of a high total radiation dose with ART for cervical cancer but is distinct as the first report of doing so with CBCT guidance. Of note, there are potential limitations to using CBCT guidance in this setting. Though CT can sufficiently delineate critical OARs [35], MRI is superior to CT in delineating the margins of cervical tumors and, as such, is an essential component of initial treatment planning in EBRT simulations and the initial fraction of brachytherapy [36]. It is therefore possible that MR-guided ART may better characterize any tumor regression that occurs during treatment, thereby allowing shrinkage of targets and potential further OAR sparing. However, given that all fractions of ART are delivered within a week, we would not anticipate significant tumor regression between each fraction and believe a pre-treatment simulation MRI should be sufficient. Our approach is also time-intensive, with a mean treatment time of 58 minutes, which can limit the ability to use this treatment modality for large volumes of patients. The ETHOS platform is continually evolving, and it is possible that further refinement of auto-contouring and plan re-optimization may reduce treatment time in the future.

## Conclusions

Herein, we illustrate a case of CBCT-guided adaptive radiation boost for a patient with cervical cancer who was ineligible for brachytherapy. We demonstrate the feasibility of our approach and the dosimetric benefits of plan adaptation in this setting. Though larger-scale and longer-term data are needed, this case demonstrates a potential approach to boost tumors in a challenging treatment setting.
